# Gender Identity and Gender Dysphoria in Autism Spectrum Disorders: A Systematic Review

**DOI:** 10.62641/aep.v54i3.2056

**Published:** 2026-06-15

**Authors:** Sara Zekri, Esperanza Navarro-Pardo, Francisco Alcantud-Marín

**Affiliations:** ^1^Department of Developmental and Educational Psychology, Universitat de Valencia, 46010 Valencia, Spain

**Keywords:** Autism spectrum disorders, gender dysphoria, gender identity, comorbidity, adults

## Abstract

**Background:**

Autism spectrum disorders (ASD) are a set of neurodevelopmental disorders characterised by social-communication deficits and repetitive behaviours. Research on gender differences in ASD has so far been limited although, recently, there has been increased interest in exploring how ASD relates to gender identity (GI) and gender disorders, such as gender dysphoria (GD); in this sense, some studies suggest a significant correlation between ASD and GD, finding that individuals with ASD more frequently exhibit GD traits compared to the general population. This article systematically reviews the relationship between ASD and GD on adults without cognitive impairment.

**Methods:**

The search was performed in the Web of Science (WOS) database, as well as in Scopus-Elsevier, PubMed, PsycInfo and Embase, with the keywords "autism" AND "gender", “autism disorder” AND “gender”, “ASD” AND “gender”, limiting it by title and published since 2013, after the publication of the DSM-5 (Diagnostic and Statistical Manual of Mental Disorders 5th Edition).

**Results:**

The screening process shows a low number of papers (12 articles) with diverse research methodologies and mostly small and convenience samples, composed of individuals from Western societies with a medium-high sociocultural background; four of them have focused on GD, four have studied GI, four both of them. The results indicate a relationship about ASD and diversity of GI, as well as a certain positive correlation between ASD and GD. Some studies also found influence of ASD on sexual orientation and on libido.

**Conclusions:**

It is suggested that ASD may influence GI formation and that may have some influence on GD. The importance of recognising diversity in GI in individuals with ASD, is emphasised for a better clinical support. This review highlights the need for further research with larger samples and more representative samples, including more gender-balanced samples, as most of the reviewed studies focused on males.

**The registration number in the International Prospective Register of Systematic Reviews PROSPERO is CRD42024360335:**

.

## Introduction

### Autism Spectrum Disorders

Autism spectrum disorders (ASD) are a complex set of neurodevelopmental disorders that are defined according to the DSM-5 (Diagnostic and Statistical Manual of Mental Disorders, Fifth Edition) [1] by two groups of symptoms: (a) communication and social interaction deficits (CSIDs) and (b) the presence of patterns of restrictive and repetitive interests, behaviours and activities (RRIBs). These symptoms appear in initial stages of development, last a lifetime, and are manifested in the most important areas of normal functioning (affective, academic, work, social, etc.), causing a clinically significant deterioration.

Over the last few decades, a considerable increase in the prevalence of ASD has been observed. In the United States, according to the latest data published by the Autism and Developmental Disabilities Monitoring (ADDM) Network, in 2020, the prevalence was 1 in 36, considering boys and girls together [2]. Regarding gender, Navarro-Pardo *et al*. [3] indicate that there is a disproportion in terms of prevalence, with a greater number of cases among men, in proportions of 3:1, 4:1 or 5:1. The differences are justified by the methodology used or by the age of the cohort included in the research, but they are consistent regardless of geographic origin, culture, ethnicity, etc. [4]. It is commonly accepted in the literature that the male to female ratio for ASD diagnoses is approximately 4:1 [5]. Furthermore, it is only in recent years that many investigations have been interested in explaining the differential gender prevalence rate in the diagnosis of ASD [3, 6].

### Gender Differences in ASD Symptomatology

Although the prevalence of ASD is higher in men than in women, the impact of gender differences has received little attention from researchers [5] and more research is needed in order to have conclusive results. Accordingly, Cariveau *et al*. [7] observed few gender differences in the clinical characteristics before treatment carried out and none in response to it; in the same line, Knutsen *et al*. [8] examined gender differences in symptoms of repetitive and restricted behaviours, finding more similarities than differences.

However, Coffman *et al*. [9] did find differences in the behavioural phenotype of ASD, men tended to exhibit more restrictive and repetitive behaviours than women. On the other hand, Wieckowski *et al*. [10] concluded that women showed greater emotional dysregulation than men, especially dysphoria and emotional intensity. Regarding the cognitive field, some studies have found that boys with ASD show a higher level of overall cognitive development than girls (although differences not remained robust longitudinally) [11], and that women with ASD have a lower IQ than men [12].

The only study to date that has identified gender differences in a purely community sample was Lawson *et al*. [13]. When longitudinally evaluating a cohort of children with autism at 24 and 48 months of age, they found no differences in overall autism severity scores, using the Autism Diagnostic Observation Schedule (ADOS) [14], nor in restricted or repetitive behaviours; however, other studies found that women had greater difficulties than men in social communication [15].

### GD, Gender Identity (GI) and Gender Self-Concept

People with autism often show other psychiatric comorbidities, especially anxiety disorders, emotional disorders, and attention deficit/hyperactivity disorder, although the prevalence varies depending on age and whether or not intellectual disability coexists; however, most published studies (including reviews) do not find GD among their results [16, 17]. This specific comorbidity (between ASD and GD) has only recently received limited attention [18].

According to the DSM-5, GD is characterised by discomfort and distress stemming from a discrepancy between one’s experienced gender and assigned gender, accompanied by a strong and persistent desire to be of a different gender [1]. It has been suggested that individuals with GD may have a higher-than-expected prevalence of ASD [19, 20]. Importantly, several case studies [21, 22, 23, 24, 25] and empirical reports [20, 26, 27] suggest an association between ASD and gender dysphoria (GD). Therefore, although autism and GD have clear phenotypic differences, some research findings suggest a potential link between the two [28].

De Vries *et al*. [20] have studied the co-occurrence of ASD and GD, and they have found that 7.8% of children and adolescents with GD met the criteria for an ASD diagnosis. These individuals were more frequently diagnosed with GI disorder not otherwise specified rather than GI disorder, indicating that their GD was considered to have an atypical quality [20].

Regarding the adult population, Jones *et al*. [26] have examined the co-occurrence of GD and ASD in a group of adults with GD using the Autism Spectrum Quotient (AQ) [29]. They have found that females with GD reported a higher mean AQ compared to typically developing (TD) females, while males with GD did not differ from TD males. Thus Jones *et al*. [26] suggested that the increased number of autistic traits among their female-to-male transsexual subjects could be explained by the Extreme Male Brain (EMB) theory of autism [30]. The EMB theory posits that autistic females are hyper-masculinised in certain cognitive and behavioural aspects due to elevated levels of foetal testosterone (fT) [31] but some applied investigations have found than ASD men showed less masculine characteristics compared than the controls [32]; so the effects of EMB on brain development could be not so straightforward and may require further research. In addition, many gender-related differences on terms of health illnesses have been found among ASD people but they have not yet investigated under the EMB theory.

Another study [27] assessing AQ traits among a clinical population diagnosed with GD, found evidences suggesting an association between ASD and GD: 7.1% of females with GD (N = 28) and 4.8% of males with GD (N = 63) met screening diagnostic cut-offs based on their AQ scores. This contrasts with findings from Jones *et al*. [26], where only females with GD showed higher AQ scores compared to typically developing individuals. Additionally, Bejerot and Eriksson [32] identified a gender-atypical pattern in their study involving 50 adults with ASD compared to 53 typically developing individuals in Sweden. They observed that males with ASD displayed fewer masculine characteristics and females with ASD exhibited fewer typical feminine traits compared to participants with normal development.

Therefore, the prevalence rates of GD and ASD indicate that both disorders are relatively uncommon, and their co-occurrence may be considered even rarer [27]. Even among those with GD who do not have a clinical autism diagnosis, many exhibit a higher number of autism traits compared to neurotypical individuals [26, 33, 34].

When it comes to gender self-concept, Williams *et al*. [35] were the first to document two cases of boys diagnosed with ASD who exhibited concurrent GI difficulties, characterised by cross-gender stereotyped interests and behaviours; other subsequent case studies have indicated this same connection (e.g., [22, 23, 24, 36]).

Furthermore, some studies have shown that ASD is overrepresented in both men and women with GI disorder [20] and, conversely, GI disorder appears to be overrepresented in individuals with ASD [37]. Additionally, bisexuality and homosexuality are reported to be more common in men with ASD compared to the general male population [38]. Higher rates of homosexuality and bisexuality have been noted among females with GD [39] and among autistic females [32, 40, 41].

The study of gender self-concept is particularly crucial for understanding disorders associated with GI difficulties, such as ASD [42]. In view of the above, given that there is greater interest in GD, gender roles and GI, as well as ASD, but that there is currently a lack of comprehensive critical reviews on GI and GD in people with ASD, this systematic review aims to address this gap by systematically analysing these variables in the selected articles.

## Methods

### Search Strategy

The protocol of this systematic review was pre-registered in the International Prospective Register of Systematic Review PROSPERO under the number CRD42024360335 (https://www.crd.york.ac.uk/PROSPERO/view/CRD42024360335).

First, a systematic search of the variables involved in ASD was conducted. The search was performed in the Web of Science (WOS) database, as well as in Scopus-Elsevier, PubMed, PsycInfo, and Embase, with the keywords ”autism” AND ”gender”, “autism disorder” AND “gender”, “ASD” AND “gender”, limiting it by title. The search was restricted to studies published since 2013, so all studies were developed after the publication of the DSM-5.

### Eligibility Criteria

The selected articles had to meet the following eligibility criteria. As an inclusion criterion, they had to refer to empirical studies, providing as much information as possible on the type of design; the study population, exposure factors, results and effect size.

Articles referring to populations under 18 years of age were excluded, as were studies with samples of autistic individuals with intellectual disabilities or other concurrent mental disorders.

### Study Selection Process

To avoid potential bias, the three researchers participated in the search for published studies. The first author conducted the initial search and shared the results with the other two researchers. At each stage of the process, the researchers verified the selection and discussed cases where there was no consensus until a list of articles for each stage was obtained. This process was repeated successively until the final sample of twelve publications was reached. Then, a total of 3397 documents were found in the initial keyword search; only articles were included and duplicates were removed, resulting in 320 items. Theoretical articles, doctoral theses, reviews, and other types of documents were excluded. In addition, the articles referring to children or adolescents under the age of 18 were excluded (93 studies).

**Fig. 1.**
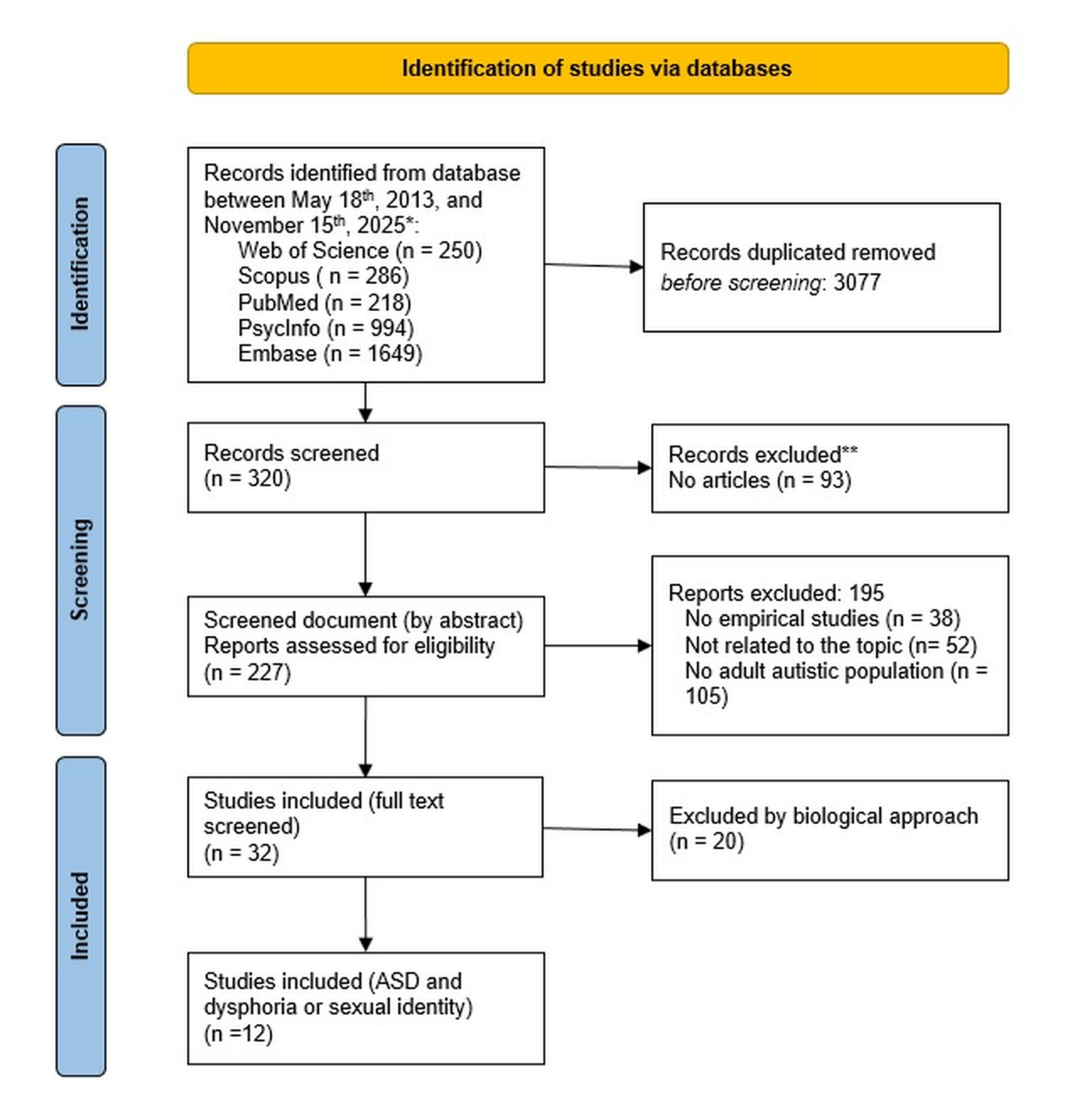
**Flow diagram summarising the article selection process**. Note: ASD, Autism spectrum disorders.

After applying all these inclusion criteria, 227 articles were selected (by abstract). Of these, only 32 met the inclusion criteria. Finally, after reading the full text, a total of 12 articles were included. The details and the complete process of selecting the bibliography are both illustrated in the PRISMA flow diagram presented below in Fig. 1.

In order to find a common thread among all the articles, it was verified that there was a relevant scientific literature on the topic of gender in autism [27, 32, 40, 43, 44]. Therefore, only the 12 articles that referred to GD or sexual identity were selected and used to prepare this article. Since the main generic scientific databases have been explored (WOS, PubMed and Scopus-Elsevier), searching other databases has not been considered.

### Assessment of the Methodological Quality of Studies

Once the studies had been selected, their methodological quality was assessed. Various tools were used for this purpose as JBI (Joanna Briggs Institute) Analytical cross-sectional studies, JBI Prevalence Studies, and JBI Qualitative Research [45], AXIS (Appraisal tool for Cross-Sectional Studies) [46], MMAT (Mixed Methods Appraisal Tool) [47], and CASP (Critical Appraisal Checklists) [48], depending on the research methodology used in each study. The results of these assessments are shown in Table 1 (Ref. [18, 27, 32, 40, 42, 43, 44, 49, 50, 51, 52, 53]).

**Table 1.** **Summary of studies on sex differences (related to GI or GD) in Autism Spectrum Disorder**.

Article	Main focus	Size and description of the sample	Methodological quality tool	Quality outcome
[32] Bejerot & Eriksson (2014)	Gender role and sexuality	ASD group: 26 males (M = 31.8, SD = 7.8), 24 females (M = 28.1, SD = 6.3) Control group: 28 males (M = 32.9, SD = 7.4), 25 females (M = 27.7, SD = 6.7) N = 103	JBI Analytical Cross-sectional Studies	Moderate-to-high
[27] Pasterski *et al*. (2014)	GD	63 male-to-female (MtF) (M = 45.47 years) 28 female-to-male (FtM) (M = 27.38 years) N = 91 transsexuals	JBI Analytical Cross-sectional Studies	Moderate-to-high
[40] George & Stokes (2018)	GI/GD	G1: 261 TD individuals (M = 30.20, SD = 11.92; 103 males and 158 females) G2: 310 ASD individuals (M = 31.01, SD = 11.37; 90 males, 219 females, and 1 intersex individual) N = 571	JBI Analytical Cross-sectional Studies	Moderate-to-high
[43] Heylens *et al*. (2018)	GD	MAB = 33; (M = 31.3 years SD = 14.7) FAB = 30; (M = 22.7 years SD = 6.5); sex ratio = 1.1:1) N = 63	JBI Prevalence Studies	Moderate
[44] Vermaat *et al*. (2018)	GD	GD sample (M = 30.20, SD = 11.57) 191 males (M = 32.46, SD = 11.99) 135 females (M = 27.02, SD = 10.18) N = 326 GD N = 174 TD	JBI Prevalence Studies	Moderate-to-high
[42] Kallitsounaki & Williams (2020a)	Gender self-concept	50 females (age range 22 to 70 years, M = 36.93, SD = 10.11) N = 101 TD N = 13 ASD	JBI Analytical Cross-Sectional Studies	Moderate-to-high
[18] Kallitsounaki & Williams (2020b)	GD	50 females (age range 22 to 70 years, M = 36.93, SD = 10.11) N = 101 TD N = 13 ASD	JBI Analytical Cross-Sectional Studies	Moderate-to-high
[49] McQuaid *et al*. (2022)	Diagnosis; Gender role; GI; Camouflaging	276 females and 226 males (aged 18–49 years) (M = 32.97, SD = 8.7) N = 502 ASD	JBI Analytical Cross-Sectional Studies	High with minor limitations
[50] Putnam *et al*. (2023)	GI	29 females, 24 NB adults, and 18 males (aged 18 to 70 years, M = 32.76, SD = 11.35) N = 71 ASD	AXIS + MMAT	Moderate-to-high + high
[51] Coleman-Smith *et al*. (2020)	GD	5 females and 5 males (M = 37.4, SD = 12.02) N = 10 ASD + GD	JBI Qualitative Research	Moderate-to-high
[52] Cooper *et al*. (2023)	GI (Transgender) and ASD	21 ASD adults (aged 18–51 years) (M = 29.1, SD = 11.5), 15 ASD adolescents (aged 13–17 years) (M = 15.7, SD = 1.28), 16 parents (M = 48, range 42–55), 16 clinicians (15.13 years qualified in profession) N = 68	CASP	High with minor limitations
[53] Shimoyama & Endo (2024)	GI (Transgender) and ASD	15 adults (3 female and 12 male), 20–49 years (M = 31.3, SD = 10.2) N = 15	CASP	High

Note: N, number; ASD, Autism Spectrum Disorders; GD, gender dysphoria; GI, gender identity; TD, typical development; M, mean age; SD, standard deviation; G1, Group 1; G2, Group 2; JBI, Joanna Briggs Institute Critical Appraisal Tool; AXIS, Appraisal Tool for Cross-Sectional Studies; MMAT, Mixed Methods Appraisal Tool; CASP, Critical Appraisal Skills Programme; MAB, Individuals assigned male at birth but who identify as female; FAB, individuals assigned female at birth but who identify as male.

## Results

Table 1 (Ref. [18, 27, 32, 40, 42, 43, 44, 49, 50, 51, 52, 53]) presents a brief description of the main characteristics of each of the 12 selected articles, including the author/s, year of publication, type of study, main implicated variables, sample as well as the methodological quality assessment tool used to evaluate each study and the result of this evaluation.

### Sample

With regard to sex, all of the studies included both men and women. However, the vast majority included more men than women [18, 27, 32, 42, 43, 44]; only three included more women than men [40, 49, 50]. Regarding sample size, the range varies between 571, from George and Stokes’ [40] largest sample, to the smallest one (N = 63) by Heylens *et al*.’ [43].

Whereas most articles included adults with autism, three of them recruited adults from the general population with GD and autistic traits, even if they did not have a diagnosis of ASD.

Concerning sample ages, all selected articles focused on adult population; the minimum age was 18 years, although some articles covered a wide age range (from 22 to 70 years) [18, 42]. Some studies reported the mean age of each sex, while others calculated the overall mean of the sample; among those reporting sex-specific means, it is noteworthy that the mean age of men was higher than that of women.

### Type of Study

There are also differences in terms of study design. In terms of methodological approach, most are quantitative [18, 27, 32, 40, 42, 43, 44, 49] but three are qualitative [51, 52, 53] and one is mixed (quantitative combined with two open-ended questions with free responses) [50]. With regard to assessment tools, most use standardised questionnaires [18, 27, 32, 40, 42, 43, 44, 49] and some use semi-structured interviews [51], but some combine both methods [50, 52, 53]. With regard to the manipulation of variables and random assignment of subjects, all are observational [42, 43, 49, 51, 52] and some also make comparisons [18, 27, 32, 40, 44, 49, 50]. Regarding temporal design, all are cross-sectional and only one also includes retrospective reviews of participants’ medical records [43]. Finally, to point out that none of the studies found used experimental methods or clinical trials.

### Variables

Regarding the variables studied, whereas five of them focused on GD [18, 27, 40, 43, 44], the remaining of the articles studied GI [50], gender self-concept [42], or gender role and sexuality [32]. The study that investigated the most variables was McQuaid *et al*. [49] which examined diagnosis, gender role and identity and camouflaging.

### Main Findings

The variety of the samples makes it difficult to abstract the findings; in the same way, the studied variables (autism, GI, GD) are classified and coded using a variety of criteria, which partially hinders the subsumption of research findings.

In respect of relationships between ASD and GI, overall, it appears a relationship between the two constructs; then, ASD set up a unique experience to the formation and consolidation of GI [40]. In this sense, masculinity traits were weaker in people with ASD than in control group while tomboyish was overrepresented amongst women with ASD [32]. Furthermore, a sample with clinically significant ASD traits showed significantly weaker both explicit and implicit GI than a sample with low ASD traits, similarly in both males and females [40], while a negative and significant association arises in general population between ASD traits and the strength of both explicit GI and implicit GI [40]. Specifically, a higher prevalence of ASD traits has been observed in transgender individuals than in the general population [27].

One study showed that GI even influences the topics of interest of people with ASD regarding their ASD condition (women and non-binary adults versus autistic men) [50].

Another of the articles focused on sex and GI effects on camouflaging, detecting higher scores in women with ASD than in men, while gender-diverse adults reported elevated camouflaging compared to cisgender adults, demonstrating that some aspects of camouflaging may have particular implications for the intersection of neurodiversity and gender diversity [49].

Regarding relationship between ASD and GD, almost all studies found a positive association between ASD/ASD traits and higher GD traits [18, 40], with almost 5% of the patients with GD showing autistic traits (measured with a standardised questionnaire) and 6% (assessed through medical records), which is sixfold higher than general population, and significantly more in men than in women [43]. Only one study [44] pointed in the opposite direction, finding the same proportion of autistic traits in people with GD than in the general population, for both males and females but, if only selecting people with GD, a positive association between ASD and GD symptoms was found; it should be noted that this study proposes a sampling methodology that is the reverse of most studies, because it compares two samples with and without GD and evaluates symptoms of autism in them. In addition, the control group (without GD) was obtained from three different samples taken from the literature.

When studied transsexual individuals with ASD, autism arose as both a barrier and a protective factor for understanding the phenomenon of GD and addressing it [51], while another study proposed a new construct in the field of mental health (pervasive social dysphoria), which would include GD as one more symptom [53].

Regarding clinicians working with people with ASD and GD in specialised gender units (adults, adolescents and parents of patients), they noticed that autistic people spoke about their gender in different ways to non-autistic people (even though they found differences among the different groups evaluated) and tried to adapt their practice to both better assessment and treatment to autistic patients with GD [52].

About relationships between ASD and sexuality, the relationship between autistic traits and sexual orientation was mediated by GD traits [40], and bisexuality were overrepresented amongst women with ASD [32], while a lower libido appeared among participants with ASD than in controls [32].

### Methods Quality and Potential Bias

Although few in number, studies show a wide variety of research methodologies, including standardised [27] and non-standardised assessment questionnaires [32, 50], self-reported questions (sometimes retrospective, about childhood, etc.) [40, 43, 44], online assessment without clinical evaluation [18, 40, 42, 49, 50], experimental tasks [18, 42], algorithms [27], qualitative questions [51, 53], interviews [52, 53], etc. Verified clinical information is only included in three studies [27, 42, 43]. Stronger methodologies with clinical and statistical reliability must be implemented.

The analyses are only descriptive, qualitative or at most correlational; in no case are prospective or predictive analyses performed. It is needed to develop studies that define dependent and independent variables, use longitudinal and cross-cultural designs, and include information obtained by clinicians. In addition, predictive analyses would be extremely useful for clinical practice.

In terms of sampling, they are not usually stratified, but rather convenience samples (volunteers, recruited through ASD-themed websites or social networks such as Facebook and Twitter, patients from mental health units, etc.) [18, 27, 32, 40, 42, 43, 49, 50, 51, 52, 53] and only six studies included case-control studies or comparisons with samples of typical development or general population [18, 27, 32, 40, 42, 43]; in one case, participation included financial remuneration [42]. Furthermore, the samples are usually small and three of them [27, 51, 53] are composed of transgender people and/or people undergoing medical treatment for gender transition. The samples are almost exclusively made up of people of Caucasian origin who have completed secondary or higher education. In short, the samples are not representative, and their results should not be generalised.

Regardless of the methodology used in the studies, in terms of the overall assessment of methodological quality, most can be rated as moderate to high (seven), only one as moderate, two as high with minor limitations, and one as high quality; the mixed methodology study obtained a double rating (high and moderate-high). This implies that the results obtained in the research found are more than acceptable.

## Discussion

### ASD and Self-Concept

Kallitsounaki and Williams [42] studied the association between ASD traits and implicit and explicit gender self-concept. Their findings indicate that individuals diagnosed with ASD often report a less pronounced masculine self-concept compared to neurotypical individuals. These results suggest that ASD may uniquely shape the development and consolidation of gender identity.

### ASD and GD

Most studies confirm a positive correlation between ASD traits and GD [18, 40, 43], with higher autistic traits associated with more pronounced GD characteristics. George and Stokes [40] found that autistic individuals showed a higher prevalence of gender dysphoric characteristics, suggesting ASD may influence gender identity development. Heylens *et al*. [43] observed an overrepresentation of autistic traits among both males and females with GD. Kallitsounaki and Williams [18] reported that the greater the ASD traits, the more GD traits appeared.

Exceptions include Pasterski *et al*. [27], who found no significant differences in AQ scores between male and female transgender individuals, and Vermaat *et al*. [44], who observed similar AQ scores between individuals referred for GD and typically developing controls. However, subgroup analyses showed higher dysphoria among those with elevated GD symptoms. Females with GD tended to present higher ASD traits than males [44], highlighting potential sex-specific mechanisms.

### Gender Identity, Roles, and Sexual Orientation

Bejerot and Eriksson [32] reported that traits associated with masculinity, such as assertiveness, leadership, and competitiveness, were less pronounced in individuals with ASD than in controls, regardless of gender. Tomboyish behaviours and bisexuality were overrepresented among women with ASD, and both males and females reported lower libido than controls. George and Stokes [40] found that higher ASD traits correlated with weaker explicit and implicit GI in both sexes. Camouflaging and compensatory behaviours were influenced by sex, GI, and timing of diagnosis. McQuaid *et al*. [49] observed higher camouflage in females with ASD and greater compensation among gender-diverse adults. Putnam *et al*. [50] showed that women and non-binary adults identified unique research priorities in autism related to gender, highlighting the importance of including diverse perspectives in research.

### Gender Diversity Representation

Women and non-binary adults are underrepresented in autism research [50]. Their perspectives and priorities regarding gender and autism topics differ from men’s, emphasizing the importance of inclusive research designs that account for gender diversity.

### Methodological Considerations and Limitations

Most studies relied on small, non-representative, or convenience samples, often including participants with higher education, Caucasian backgrounds, and predominantly male participants [18, 27, 32, 40, 42, 43, 49, 50, 51, 52, 53]. As for data collection methods, there is a wide variety; self-report tools, retrospective assessments, and qualitative methods were predominant, with few studies including clinical verification [27, 42, 43]; cross-cultural perspectives, as well as longitudinal studies and clinical trials are lacking. The heterogeneity of samples and variables, the co-occurrence of categorical (GD) and dimensional (ASD) constructs, and potential confounding variables (e.g., social pressure, camouflage, cognitive style differences) limit generalizability. These factors highlight the complexity of the ASD-GI/GD relationship and the need for careful methodological design in future research. Despite all this, the overall assessment of methodological quality is moderate to high, meaning that the results achieved in the research found are highly acceptable.

Future studies should include larger, gender-balanced, and culturally diverse samples, longitudinal designs, multimodal assessments, and follow-up of individuals diagnosed with both ASD and GD [43, 54, 55, 56, 57]. Exploration of genetic, neurophysiological, and neuroimaging correlates is also warranted [58].

### Clinical Implications

Clinicians working with individuals with ASD should be aware of gender diversity within this population and tailor assessments and interventions accordingly [52]. Specialized training can improve understanding of how autism and GD intersect, facilitating adjustments in clinical practice. Accurate diagnosis requires differentiating between ASD and GD symptoms, and consideration of co-occurring psychiatric disorders is essential to avoid misattribution or underestimation of needs [43]. Applied research, expert consensus, and clinical guidelines on ASD and GD co-occurrence are needed to inform assessment and intervention strategies [59]. Improving awareness, applying specific assessment tools, and providing medical and psychological support may enhance socio-sexual functioning and mental well-being [40].

## Conclusions

This systematic review indicates that ASD traits are frequently associated with GD, gender identity, and related constructs. Individuals with ASD experience unique interactions between their neurodevelopmental characteristics and gender self-concept, with women and non-binary adults showing distinct patterns.

Despite the limited number of studies, small and non-representative samples, and predominance of self-report methods, the findings underscore the clinical relevance of recognizing gender diversity within the ASD population. Tailored assessment and intervention strategies are necessary to support socio-sexual functioning and mental well-being.

Future research should prioritize gender-balanced, culturally diverse, and longitudinal studies to clarify the co-occurrence of ASD and GD and to guide evidence-based clinical practice.

## Availability of Data and Materials

Fully anonymised data will be made available on request to the corresponding author.

## Supplementary Material

Supplementary material associated with this article can be found, in the online version, at https://doi.org/10.62641/aep.v54i3.2056.
